# Lysine demethylase 5C inhibits transcription of prefoldin subunit 5 to activate c-Myc signal transduction and colorectal cancer progression

**DOI:** 10.1186/s10020-023-00775-7

**Published:** 2024-01-12

**Authors:** Fulong Yu, Liang Li, Yimei Gu, Song Wang, Lianbang Zhou, Xiaohu Cheng, Heng Jiang, Yang Huang, Yingfeng Zhang, Wenbao Qian, Xianghua Li, Zhining Liu

**Affiliations:** 1 Department of General Surgery, The Second Affiliated Hospital of Anhui Medical University, Hefei, 230601 Anhui People’s Republic of China; 2 Emergency ICU, The First Affiliated Hospital of Anhui Medical University, Hefei, 230000 Anhui People’s Republic of China; 3 Department of General Surgery, The Second Affiliated Hospital of Anhui Medical University, No. 678 Furong Road, Hefei, 230601 Anhui People’s Republic of China; 4 Department of Molecular Pathology, Hefei Da’an Medical Laboratory Co., Ltd., Hefei, 230012 Anhui People’s Republic of China

**Keywords:** Lysine demethylase 5C, Prefoldin subunit 5, Colorectal cancer, Autophagy, Apoptosis

## Abstract

**Background:**

Lysine demethylase 5C (KDM5C) has been implicated in the development of several human cancers. This study aims to investigate the role of KDM5C in the progression of colorectal cancer (CRC) and explore the associated molecular mechanism.

**Methods:**

Bioinformatics tools were employed to predict the target genes of KDM5C in CRC. The expression levels of KDM5C and prefoldin subunit 5 (PFDN5) in CRC cells were determined by RT-qPCR and western blot assays. The interaction between KDM5C, H3K4me3, and PFDN5 was validated by chromatin immunoprecipitation. Expression and prognostic values of KDM5C and PFDN5 in CRC were analyzed in a cohort of 72 patients. The function of KDM5C/PFDN5 in c-Myc signal transduction was analyzed by luciferase assay. Silencing of KDM5C and PFDN5 was induced in CRC cell lines to analyze the cell malignant phenotype in vitro and tumorigenic activity in nude mice.

**Results:**

KDM5C exhibited high expression, while PFDN5 displayed low expression in CRC cells and clinical CRC samples. High KDM5C levels correlated with poor survival and unfavorable clinical presentation, whereas elevated PFDN5 correlated with improved patient outcomes. KDM5C mediated demethylation of H3K4me3 on the PFDN5 promoter, suppressing its transcription and thereby enhancing the transcriptional activity of c-Myc. KDM5C knockdown in CRC cells suppressed cell proliferation, migration and invasion, epithelial-mesenchymal transition, and tumorigenic activity while increasing autophagy and apoptosis rates. However, the malignant behavior of cells was restored by the further silencing of PFDN5.

**Conclusion:**

This study demonstrates that KDM5C inhibits PFDN5 transcription, thereby activating c-Myc signal transduction and promoting CRC progression.

## Background

In 2020, colorectal cancer (CRC) accounted for over 1.9 million new cases globally and resulted in around 935,000 deaths, making it one of the most prevalent solid tumors, contributing to 1 in 10 cancer cases and deaths worldwide (Sung et al. 2021). Risk factors for CRC development include genetic factors, hyperlipidemia, obesity, alcohol consumption, smoking, bad dietary habits, and sedentary lifestyle (O'Sullivan et al. 2022; Vargas and Thompson 2012). Despite optimal treating strategies, the overall 5-year survival rate of CRC patients in China remains unfavorable at 31%, largely due to the late diagnosis, recurrence, and metastasis (Zhang et al. 2018). Identifying key molecules implicated in the tumor progression is crucial for developing novel therapeutic options for CRC.

The development of cancer cells results from the interplay between genetic and epigenetic modifications, creating a complex mixture of changes that drive cancer initiation and progression (Porcellini et al. 2018). This is particularly true for CRC, where a significant epigenetic component plays a critical role in the early stages of malignant transformation, preceding other genetic alterations (Lao and Grady 2011; Porcellini et al. 2018). Histone methylation, specifically lysine and arginine residues on histone tails, is a complex and subtle chromatin modification closely linked to tumorigenesis (Audia and Campbell 2016; Michalak et al. 2019). Lysine demethylase 5C (KDM5C), also known as JARID1C, is a member of the lysine demethylase family that primarily catalyzes the removal of trimethylation of histone H3 lysine 4 (H3K4me3), an active transcriptional marker, leading to transcriptional repression (Dalgliesh et al. 2010; Guo and Zhang 2017; Qi et al. 2021). While KDM5C has been identified as a tumor suppressor in clear cell renal cell carcinoma (Niu et al. 2012) and human papillomavirus-related cancers (Chang et al. 2019), it has been reported as a tumor driver in prostate cancer by influencing the epithelial-mesenchymal transition (EMT)-related signaling pathways and transcription factors (Lemster et al. 2022). However, the function of KDM5C in CRC and its substrate molecules remain largely unknown.

In this study, we employed several bioinformatics systems and identified prefoldin subunit 5 (PFDN5) as a candidate downstream target of KDM5C. PFDN5, also known as Myc-modulator 1, is reported as a binding protein of the protooncogene c-Myc, influencing its transcriptional activity and theoretically playing a tumor-suppressive function (Ariga 2015; Fujioka et al. 2001; Mori et al. 1998). c-Myc plays a central role in the reprogramming, proliferation, EMT, metastasis, and chemoresistance of various cancer cells, making it a significant transcription factor involved in these processes (Fatma et al. 2022; Zhong et al. 2021). Additionally, c-Myc overexpression has been causally correlated with the inhibition of lysosomal and autophagic function in pluripotent stem cells and cancer, potentially serving as a hallmark of malignant transformation (Annunziata et al. 2019). Autophagy, an evolutionarily conserved process, scavenges and recycles senescent or damaged organelles and biological macromolecules through lysosomal degradation, maintaining cellular homeostasis (Hu et al. 2022). This process entails the upregulation of LC3B protein, a double-membrane component of the autophagosome, the formation of autophagosome, and the fusion of autophagosome with the lysosome (Han et al. 2019). Autophagy's role in tumor progression, including CRC, remains debated, (Long et al. 2020), with its exact function across various contexts and stages yet to be fully understood. In summary, this study aims to validate the interaction between KDM5C and PFDN5 and elucidate their roles in c-Myc activation, as well as their impact on the proliferation, autophagy, apoptosis, EMT, and metastasis of CRC cells.

## Methods

### Ethical approval

The study was approved by the Ethics Committee of the Second Affiliated Hospital of Anhui Medical University and conducted in compliance with the *Declaration of Helsinki*. Written informed consent form was obtained from each eligible patient. The animal study protocol was approved by the Animal Ethics Committee of the institute, and all procedures were performed in accordance with the Guide for the Care and Use of Laboratory Animals (NIH, Bethesda, Maryland, USA).

### Clinical sample collection

A cohort of 72 patients clinically diagnosed with CRC at the Second Affiliated Hospital of Anhui Medical University from June 2017 to December 2018 were enrolled in this study. Tumor tissues and adjacent tissues (located over 5 cm from the lesion site) were collected during surgery and subsequently stored at -80℃ or embedded in paraffin. A postoperative follow-up spanning three years was conducted, during which recurrence was monitored through imaging examination systems (chest X-ray and CT), gastrointestinal endoscopy biopsy, and telephone follow-up. The patients' recurrence-free survival (RFS) was recorded. The characteristics of all patients are detailed in Table 1.

**Table 1:** Correlations between KDM5C and PFDN5 expression and clinicopathologic features in patients with CRC

Features	KDM5C expression		*p* value	PFDN5 expression		*p* value
	High (n = 34)	Low (n = 38)		High (n = 33)	Low (n = 39)	
Age (years)						
< 65	17	21	0.8134	21	17	0.1032
≥ 65	17	17		12	22	
Sex						
Male	20	19	0.4861	15	24	0.2359
Female	14	19		18	15	
TNM stage						
T1-2	1	12	0.0017	12	1	0.0003
T3-4	33	26		21	38	
LNM						
Absent	11	26	0.0043	23	14	0.0051
Present	23	12		10	25	

The clinical data were analyzed using Fisher's exact test. A *p* value less than 0.05 was indicative of statistical significance. *KDM5C* lysine demethylase 5C, *PFDN5* prefoldin subunit 5, *CRC* colorectal cancer, *TNM* tumor node metastasis, *LNM* lymph node metastasis

### Cell culture and treatment

Human CRC cell lines, HCT116 (CCL-247) and SW480 (CCL-228), were obtained from American Type Culture Collection (ATCC, Manassas, VA, USA). The normal colon cell line, NCM460 (CC-Y1550), was procured from EK-Bioscience Biotechnology Co., Ltd. (Shanghai, China). Cells were cultured in Dulbecco's modified Eagle's medium (DMEM, Thermo Fisher Scientific Inc., Waltham, MA, USA) supplemented with 10% fetal bovine serum (FBS) and cultured at 37℃ with 5% CO_2_ in a humidified atmosphere. Short hairpin (sh) RNAs targeting KDM5C and PFDN5 were designed and synthesized by GenePharma Co., Ltd. (Shanghai, China). These shRNAs were cloned to interference vector pGPU6/GFP/Neo, respectively. Lentiviral vectors were generated by co-transfecting the interference agent with VSVG and D8.9 packaging vectors into 293 T cells (CRL-3216; ATCC, Manassas, USA) using Lipofectamine 3000. After 48 h, lentiviral vectors were collected and used to infect the HCT116 and SW480 cells. The infected cells were incubated for an additional 48 h under 5% CO_2_ at 37 ℃ to establish cell lines with stable inhibition of KDM5C or co-inhibition of KDM5C and PFDN5. To rule out the potential impact of autophagy on apoptosis, cells with stable KDM5C inhibition were treated with 10 nM chloroquine (CQ, Sigma-Aldrich, USA) for 12 h prior to apoptosis detection using flow cytometry.

### Chromatin immunoprecipitation (ChIP)-quantitative polymerase chain reaction (qPCR)

The binding between KDM5C and PFDN5 promoter was assessed through the ChIP-qPCR assay. In short, CRC cells were crosslinked in 1% formaldehyde and then ultrasonicated on ice to generate DNA fragments. The resulting product was incubated with anti-KDM5C (1:50, ab264168, Abcam Inc., Cambridge, MA, USA), anti-H3K4me3 (1:50, ab8580, Abcam) or the control immunoglobulin G (IgG) at 4 ℃ overnight. Subsequently, the immunoprecipitated complexes were eluted and de-crosslinked with NaCl solution overnight. Chromatin DNA was recovered from the precipitate, dissolved in deionized water, and analyzed using qPCR.

### Immunohistochemistry (IHC)

The collected tissue samples were fixed in 4% paraformaldehyde overnight, embedded in paraffin, and cut into 5-μm sections. Following this, the sections were dewaxed, rehydrated in alcohol, boiled in citric acid for 10 min of antigen retrieval, and blocked in normal goat serum for 1 h. The prepared sections were incubated with antibodies against KDM5C (1:200, PA5-115462, Thermo Fisher Scientific, Rockford, IL, USA), PFDN5 (1:200, ab129116, Abcam), and cleaved-caspase-3 (1:200, PA5-114687, Thermo Fisher Scientific) at 4 ℃ overnight, followed by incubation with horseradish peroxidase (HRP)-conjugated IgG (1:2,000, ab6721, Abcam) at room temperature for 1 h. After color development using the DAB solution and nuclei staining with hematoxylin, the sections were observed under a microscope to evaluate the presence of positive cells.

### Reverse transcription-qPCR (RT-qPCR)

Total RNA from tissues and cells was extracted using the PureLink RNA Mini Kit (Thermo Fisher Scientific). Subsequently, the first-strand cDNA was synthesized using the PrimeScrip RT kit (Takara Holdings Inc., Kyoto, Japan). The qPCR was then conducted using TB Green Premix Ex Taq (Tli RNase H Plus) (Takara) on an ABI 7200 real-time PCR system (Applied Biosystems, Inc., Carlsbad, CA, USA). Glyceraldehyde-3-phosphate dehydrogenase (GAPDH) was used as the endogenous loading for KDM5C and PFDN5 mRNA. Relative gene expression was determined by the 2^−ΔΔct^ method. The primer sequences are as follows: KDM5C: (F) 5′-ACTGCTGACCATTGCTGAACGC-3′, (R) 5′-CCTCCTTGAGAGCCTGGATGTT-3′; PFDN5: (F) 5′-TGTGGAAGCCAAGGACTGTCTG-3′, (R) 5′-GAGCACGTGTTCCACATCATGC-3′; GAPDH: (F) 5′-GTCTCCTCTGACTTCAACAGCG-3′, (R) 5′-ACCACCCTGTTGCTGTAGCCAA-3′.

### Cell counting kit-8 (CCK-8) method

Cell viability was examined using a CCK-8 kit (Dojindo Laboratories, Kumamoto, Japan). In short, stably transfected cells were seeded in 96-well plates at 5,000 cells per well with three duplicated wells set for each group. The cells were cultured at 37 ℃ with 5% CO_2_ for 24, 48, and 72 h, respectively. Following the incubation period, each well was treated with 10 μL CCK-8 solution for an additionally 2 h at 37 ℃. The optical density value at 450 nm was read using a microplate reader.

### Transwell assays

Cell migration and invasion were assessed using Transwell assays. Briefly, 5 × 10^4^ cells resuspended in 200 μL serum-free medium were added to the apical chambers of Transwell inserts. For invasion detection, the chambers were pre-coated with Matrigel (BD Biosciences, San Jose, CA, USA). The basolateral chambers were filled with 10% FBS-supplemented DMEM. The Transwell chambers were then placed in a 37 ℃ incubator with 5% CO_2_ for 24 h. Migration of cells was examined in a similar manner, excluding Matrigel pre-coating. Cells that invaded or migrated to the lower membranes were washed, soaked in 95% ethanol for 10 min, stained with 0.1% crystal violet for 10 min, and observed under a microscope. Representative images were captured using an inverted microscope (Nikon Instruments Inc., Tokyo, Japan).

### Dual-luciferase reporter gene assay

The Myc luciferase reporter plasmid, utilized for detecting transcriptional activity of c-Myc, was supplied by YEASEN Biotechnology (Shanghai, China). The Myc response element sequence was GGCCTAACTGGCCGGTACCGCTAGCCTCGATCACGTGCACGTGCACGTGCACGTGGCGCGTAGATCTGCAGAAGCTTAGACACTAGAGGGTATATAATGG. The plasmid was transfected into CRC cells with stable KDM5C/PFDN inhibition using Lipofectamine 3000. After 48 h, the luciferase activity in cells was examined using the dual luciferase reporter kit to evaluate the transcriptional activity of c-Myc.

### Autophagosome detection

The formation of autophagosomes was assessed through immunofluorescence staining. The CRC cells were fixed in 4% paraformaldehyde for 20 min, permeabilized with 0.2% Triton X-100 for 5 min, and subsequently blocked with normal goat serum for 1 h. Following this, the cells were incubated with anti-LC3B (1:500, #3868, Cell Signaling Technology, Beverly, MA, USA) at 4 ℃ overnight, followed by incubation with IgG H&L (Alexa Fluor® 555) (1:1,000, ab150078, Abcam) at room temperature for 2 h. Subsequently, the cells were stained with 4',6-diamidino-2-phenylindole (Solarbio) for 10 min. The relative staining intensity of LC3B in the cells was then examined using a confocal microscope (Nikon Instruments Inc.).

### Western blot (WB) analysis

Total protein from the cells was extracted using radio-immunoprecipitation assay cell lysis buffer (Cell Signaling Technology) supplemented with the protease inhibitor (Roche Ltd, Basel, Switzerland). The protein concentration was determined using a bicinchoninic acid kit (Thermo Fisher Scientific). Equal amounts of protein samples (30 μg) were separated by 10% sodium dodecyl sulfate–polyacrylamide gel electrophoresis and transferred onto polyvinylidene fluoride membranes (Millipore, Billerica, MA, USA). Following a 1-h block in 5% non-fat milk at room temperature, the membranes were incubated with the primary antibodies against KDM5C (1:1,000, ab264168, Abcam), PFDN5 (1:1,000, ab129116, Abcam), ATG7 (1:1,000, ab52472, Abcam), P62 (1:1,000; NBP1-48320, Novus Biologicals, Littleton, CO, USA), Beclin1 (1:1,000, #3495, Cell Signaling Technology), LC3 (1:1,000; #3868, Cell Signaling Technology), Vimentin (1:1,000, NBP1-31327, Novus Biologicals), N-cadherin (1:1,000, NBP2-19457, Novus Biologicals), GAPDH (1:1,000; ab181602, Abcam) at 4 °C overnight. Subsequently, the membranes were incubated with the HRP-conjugated IgG (1:20,000, ab6721, Abcam) at room temperature for 2 h. Protein blots were developed using enhanced chemiluminescence reagent and analyzed using Image J.

### Flow cytometry

Apoptosis of CRC cells was assessed using the Annexin V-fluorescein isothiocyanate (FITC)/propidium iodide (PI) kit (YEASEN Biotechnology). Briefly, cells were washed and resuspended in 500 μL binding buffer to achieve a concentration of 1 × 10^6^ cells/mL. Subsequently, the cells were stained with Annexin V-FITC and PI in the dark at room temperature for 15 min. After staining, each tube received an additional 400 μL 1 × binding buffer thereafter, and the samples were promptly analyzed by the flow cytometer (BD Biosciences, Franklin Lakes, NJ, USA).

### Xenograft tumors in nude mice

Thirty BALB/c nude mice (6 weeks old) were procured from Vital River Laboratory Animal Center (Beijing, China) and were housed in facilities with a 12-h light/dark cycle, provided with ad libitum access to food and water. HCT116 and SW480 cells (1 × 10^7^) stably transfected with sh-KDM5C or sh-PFDN5 were resuspended in 100 μL serum-free medium and subcutaneously injected into the mice at the armpit site. The volume (V) of xenograft tumors was measured every 7 d using the formula: V = (major axis) × (minor axis)^2^/2. The animals were euthanized on the 28th d through intraperitoneal injection of 1% pentobarbital sodium (150 mg/kg). The tumor tissues were weighed and harvested for subsequent analysis.

### Statistical analysis

Data analysis was conducted using Prism 8.0.2 (GraphPad, La Jolla, CA, USA). For cellular experiments, a minimum of three biological replicates were performed. Measurement data are expressed as the mean ± standard deviation. Differences were assessed using the *t* test for two groups or one- or two-way analysis of variance (ANOVA) and Tukey's multiple test for more than two groups. Survival analysis was performed using the Log-rank (Mantel-Cox) test). Correlation between variables was assessed through Pearson's correlation analysis. Clinical data were analyzed using Fisher's exact test. A *p* value less than 0.05 was indicative of statistical significance.

## Results

### KDM5C regulates the transcription of PFDN5 through histone demethylation.

**Fig. 1:**
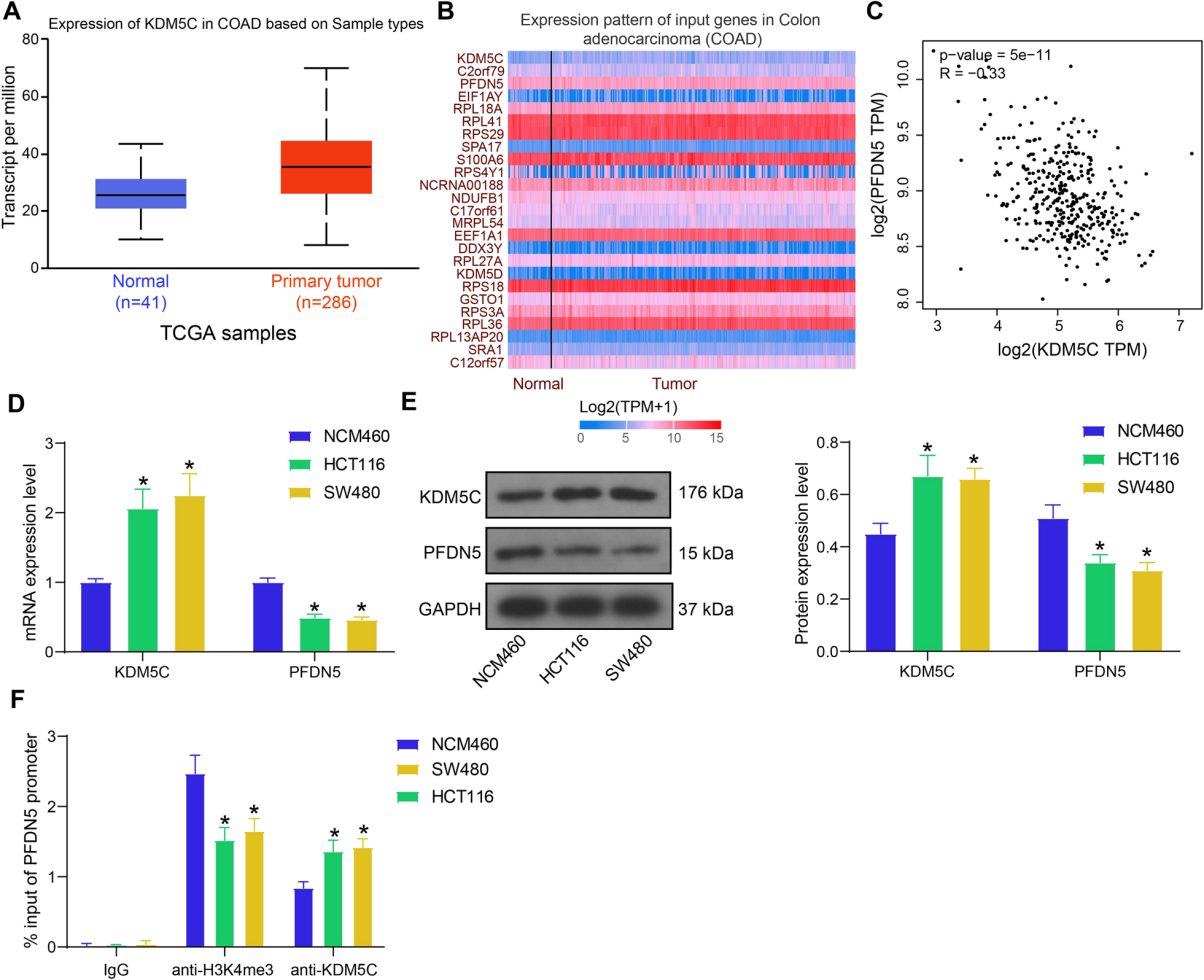
KDM5C regulates the transcription of PFDN5 through histone demethylation. **A** KDM5C expression in the CRC transcriptome database in the UALCAN system; **B** genes exhibiting negative correlation with KDM5C in CRC in the UALCAN system (ranked by correlation coefficient, with the top 25 genes displayed); **C** a negative correlation between KDM5C and PFDN5 in CRC in the GEPIA system; D-E, RT-qPCR **D** and WB analysis **E** to detect the mRNA and protein levels of KDM5C and PFDN5 in the CRC cells (HCT116 and SW480) and normal NCM460 cells (two-way ANOVA); **F** binding relationship of KDM5C and H3K4me3 with the PFDN5 promoter in the CRC cells and normal NCM460 cells validated by the ChIP-qPCR assay (two-way ANOVA). Three biological replicates were performed. *p < 0.05 vs. NCM460/IgG

Analysis of the CRC transcriptome database in the UALCAN system (https://ualcan.path.uab.edu/cgi-bin/ualcan-res.pl) revealed elevated expression of KDM5C in CRC (Fig. 1A). Given that KDM5C typically inhibits target gene expression by demethylating histones, we further examined genes exhibiting a negative correlation with KDM5C in CRC in the UALCAN system (Fig. 1B). PFDN5, a known negative regulator of c-Myc and thus a potential tumor suppressor (Ariga 2015), exhibiting a remarkable negative correlation with KDM5C expression, ranking second in correlation coefficient, was consequently chosen for subsequent analysis. Similarly, a significant inverse correlation between KDM5C and PFND5 in CRC was found in the Gene Expression Profiling Interactive Analysis (GEPIA) system (http://gepia.cancer-pku.cn/index.html) (Fig. 1C).

In vitro evaluation of KDM5C and PFDN5 expression in cells revealed that both the mRNA and protein levels of KDM5C were higher, whereas the levels of PFDN5 were lower in the HCT116 and SW480 cells than in normal colon cells NCM460 (Fig. 1D, E). Notably, ChIP-qPCR assays demonstrated an increase in the KDM5C enrichment level whereas a decrease in H3K4me3 enrichment level on the PFDN5 promoter in the HCT116 and SW480 cells compared to NCM460 cells (Fig. 1F).

### Expression levels and prognostic values of KDM5C and PFDN5 in CRC patients

**Fig. 2:**
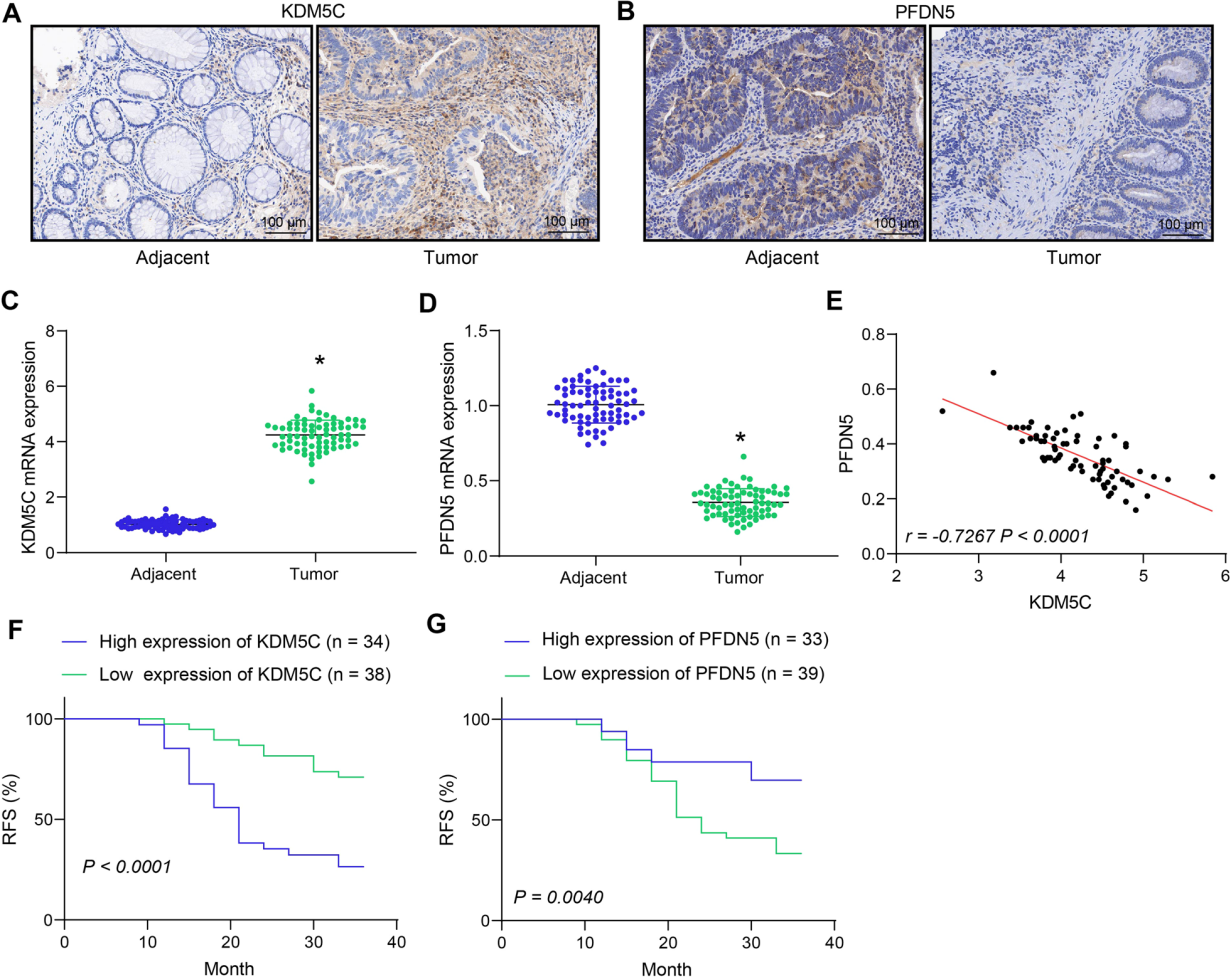
Expression levels and prognostic values of KDM5C and PFDN5 in CRC patients. **A**, **B** IHC assay to detect the protein levels of KDM5C **A** and PFDN5 **B** in CRC tissues and the adjacent normal tissues; **C**, **D** RT-qPCR to detect the mRNA levels of KDM5C **C** and PFDN5 **D** in CRC tissues and the adjacent normal tissues (n = 72) (paired *t* test); E, a negative correlation between KDM5C and PFDN5 mRNA expression in CRC tissues (n = 72) (Pearson's correlation analysis); **F**–**G**, correlations of KDM5C **F** and PFDN5 **G** expression with the RFS of patients (Log-rank (Mantel-Cox) test). *p < 0.05

The expression levels of KDM5C and PFDN5 were then assessed in the clinical samples.The IHC assay revealed a higher proportion of KDM5C-positive cells and a lower proportion of PFDN5-positive cells in CRC tissues compared to adjacent normal tissues (Fig. 2A, B). Consistent trends were observed at the mRNA levels based on the RT-qPCR results (Fig. 2C, D), and an inverse correlation between KDM5C mRNA with PFDN5 mRNA was evident in the CRC tissues (Fig. 2E). Patients were categorized into high and low expression groups based on the mean mRNA expression. Survival analysis demonstrated a significant association between high KDM5C and shorter RFS, whereas high PFDN5 expression was associated with longer RFS in patients (Fig. 2F, G). The relationships between KDM5C or PFDN5 expression and the clinical pathological features of CRC patients were further analyzed. As shown in Table 1, KDM5C expression was found to be positively correlated with lymph node metastasis and advanced tumor node metastasis (TNM) staging, while PFDN5 expression correlated negatively with both. No specific correlations were identified with age or gender in relation to gene expression. These results suggest that KDM5C may promote the progression of CRC, while PFDN5 may exert an opposite effect.

### KDM5C/PFDN5 regulates viability of CRC cells and affects c-Myc signal transduction

**Fig. 3:**
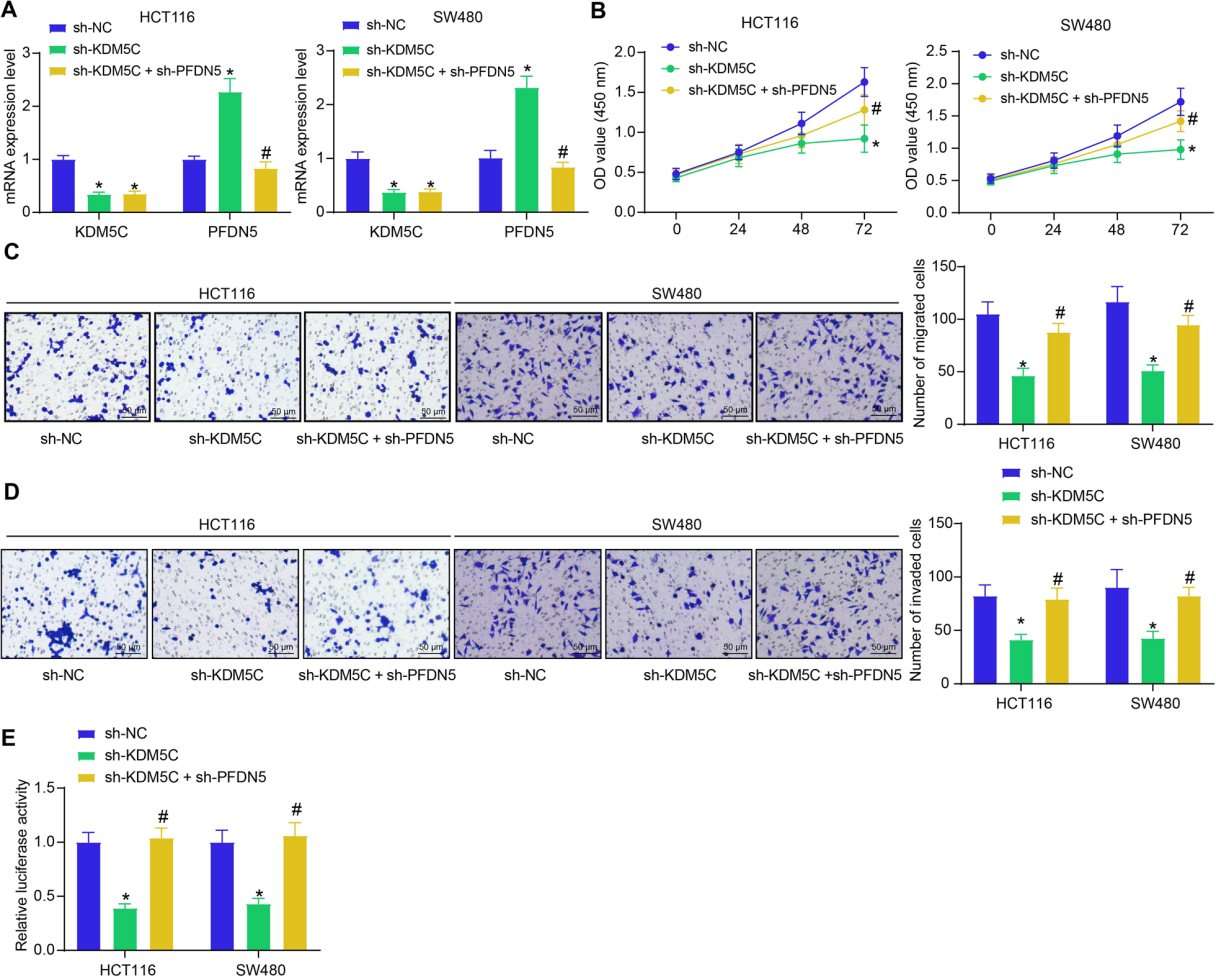
KDM5C/PFDN5 regulates viability of CRC cells and affects c-Myc signal transduction. **A** RT-qPCR to analyze the expression of KDM5C and PFDN5 in HCT116 and SW480 cells after shRNA transfection examined by (two-way ANOVA); **B** CCK-8 assay to analyze the proliferation of the cells examined by the (two-way ANOVA); **C**, **D** Transwell assays to analyze migration **C** and invasion abilities **D** of HCT116 and SW480 cells (two-way ANOVA); **E** dual luciferase assay to examine the transcriptional activity of c-Myc in the HCT116 and SW480 cells (two-way ANOVA). Three biological replicates were performed. *p < 0.05 vs. the sh-NC group; #*p* < 0.05 vs. the sh-KDM5C group

To unravel the biological functions of KDM5C and PFDN5 in CRC, HCT116 and SW480 cells with stable KDM5C inhibition alone or with co-inhibition of KDM5C and PFND5 were induced using lentivirus-carried shRNAs. As anticipated, KDM5C inhibition led to an upregulation of PFDN5 (Fig. 3A). The CCK-8 results showed that the proliferation of CRC cells was significantly reduced after KDM5C silencing but restored after further knockdown of PFDN5 (Fig. 3B). Similarly, the migration and invasion of cells, according to the Transwell assays, were suppressed by the KDM5C knockdown but recovered upon further PFDN5 downregulation (Fig. 3C, D).

As mentioned earlier, PFDN5 has been reported as a binding protein of c-Myc that affects the transcriptional activity of c-Myc. Therefore, the Myc luciferase reporter plasmid was transfected into cells transfected with sh-KDM5C and sh-PFND5. The luciferase assay revealed that the transcriptional activity of c-Myc was suppressed by KDM5C knockdown but restored after PFND5 silencing (Fig. 3E).

### KDM5C/PFDN5 regulates autophagic flux in CRC cells

**Fig. 4:**
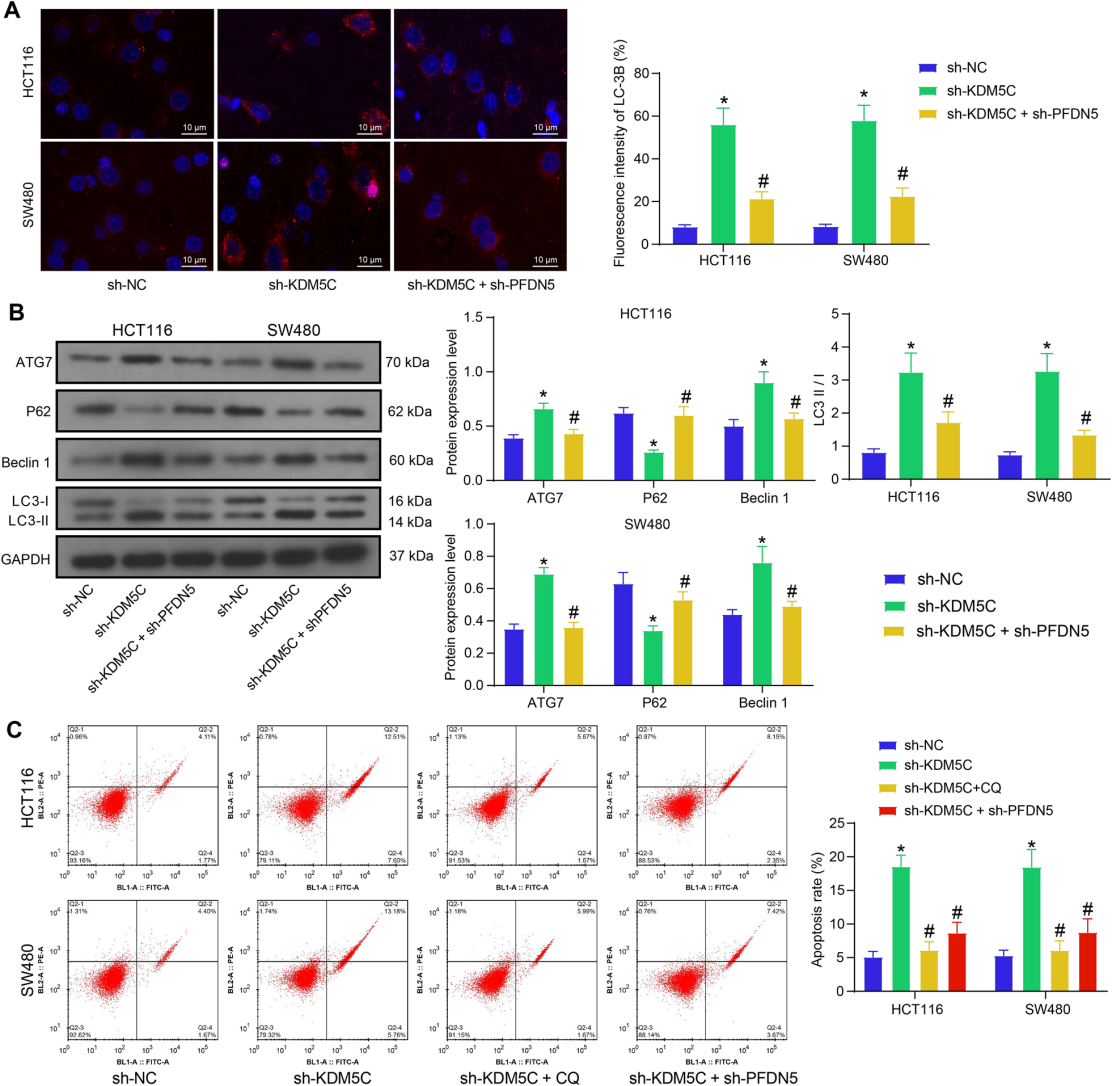
KDM5C/PFDN5 regulates autophagic flux in CRC cells. **A** immunofluorescence staining to examine the LC-3B expression in the CRC cells (two-way ANOVA); **B** WB analysis to analyze the protein levels of ATG7, Beclin1, P62, and LC3-II/LC3-I (two-way ANOVA); **C** flow cytometry to analyze the apoptosis of CRC cells (two-way ANOVA). Three biological replicates were performed. *p < 0.05 vs. the sh-NC group; #*p* < 0.05 vs. the sh-KDM5C group

Considering the reported association between c-Myc signal transduction and autophagy inhibition during cancer progression (Annunziata et al. 2019), we hypothesized that KDM5C/PFDN5 might influence the autophagy activity of CRC cells. Immunofluorescence staining showed that the intensity of LC-3B fluorescence in the cells was increased after KDM5C silencing but reduced after additional PFDN5 knockdown (Fig. 4A). Similarly, the WB analysis of autophagy-related marker proteins indicated an augmentation in autophagosome formation after KDM5C silencing. This was evident from increased protein levels of ATG7, Beclin1, and LC3-II/LC3-I, accompanied by a decline in the autophagy substrate P62 (Fig. 4B). These changes were reversed by the knockdown of PFDN5. Autophagy may induce apoptosis in cancer cells. Furthermore, we analyzed the apoptosis of CRC cells under the conditions of KDM5C/PFDN5 silencing or CQ treatment. The flow cytometry showed that the knockdown of KDM5C increased the apoptosis of HCT116 and SW480 cells. However, the cell apoptosis was reduced by further treatment of CQ or sh-PFDN5 (Fig. 4C). This evidence indicates that the KDM5C-mediated apoptosis of CRC cells might be linked to the increased autophagic flux.

### KDM5C/PFDN5 affects tumorigenesis of CRC cells in vivo

**Fig. 5:**
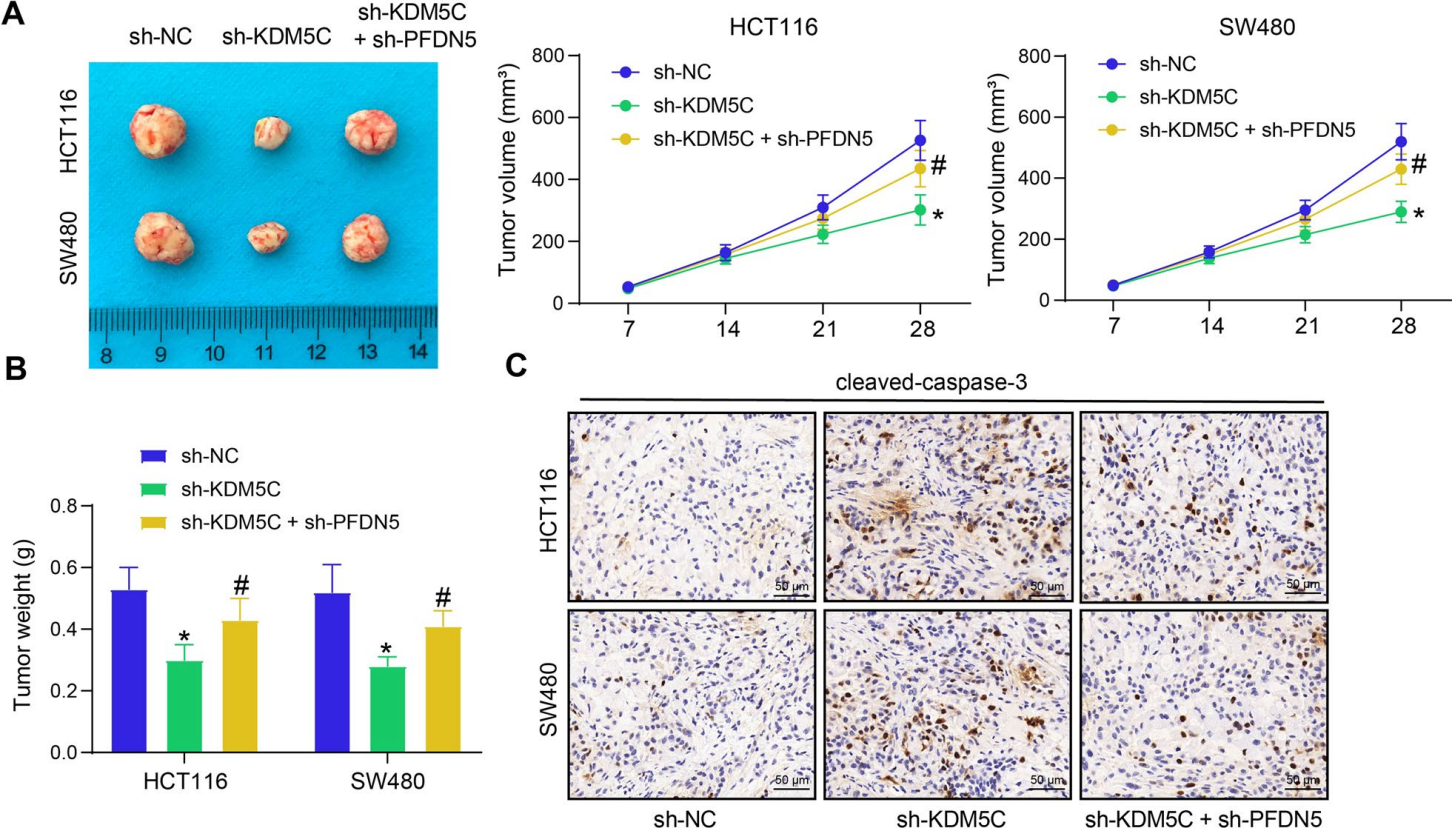
KDM5C/PFDN5 affects tumorigenesis of CRC cells in vivo. **A** representative images of xenograft tumors formed by HCT116 and SW480 cells on day 28 and the volume change of tumors during this period (two-way ANOVA); **B** weight of the xenograft tumors on day 28 (two-way ANOVA); **C**, activation of caspase-3 (cleaved-caspase-3) in tumor tissues examined by IHC. In each group, n = 5. *p < 0.05 vs. the sh-NC group; #*p* < 0.05 vs. the sh-KDM5C group

HCT116 and SW480 cells stably transfected with sh-KDM5C and sh-PFDN5 were subcutaneously injected into mice to induce xenograft tumors. Notably, the downregulation of KDM5C in cells reduced the growth rate and volume of tumors in mice, while further downregulation of PFDN5 restored the growth rate (Fig. 5A). On day 28, the mice were sacrificed, and the tumors were collected and weighed. Similarly, the tumor weight was significantly reduced when KDM5C was silenced but enhanced when PFDN5 was further knocked down (Fig. 5B). Additionally, the IHC assay revealed that the intensity of cleaved-caspase-3 staining, a marker of apoptosis, in tumor tissues was enhanced after KDM5C silencing but weakened upon PFDN5 downregulation (Fig. 5C).

### KDM5C/PFDN5 affects EMT in CRC

**Fig. 6:**
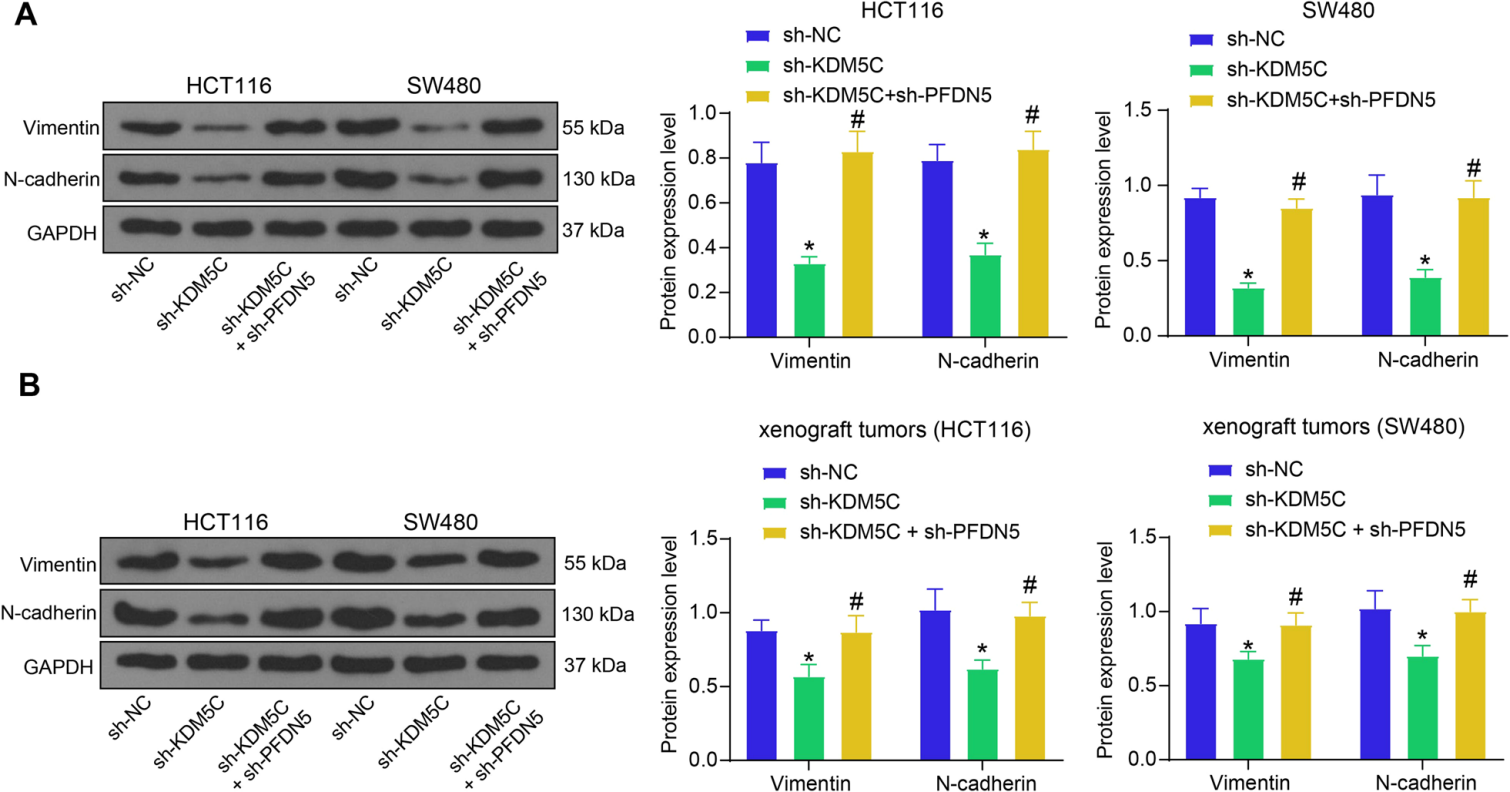
KDM5C/PFDN5 affects EMT in CRC. **A** protein levels of Vimentin and N-cadherin in HCT116 and SW480 cells determined by WB analysis (two-way ANOVA); **B** protein levels of Vimentin and N-cadherin in xenograft tumors determined by western blot analysis (two-way ANOVA). For cellular experiments, three biological replicates were performed. For animal studies, n = 5 in each group. *p < 0.05 vs. the sh-NC group; #*p* < 0.05 vs. the sh-KDM5C group

c-Myc has also reportedly been associated with increased EMT in tumors. Therefore, the role of KDM5C/PFDN5 in the EMT activity in CRC was further analyzed. The WB analysis of EMT-related proteins showed that the expression of mesenchymal markers Vimentin and N-cadherin was decreased in CRC cells upon KDM5C knockdown. These changes were reversed by the PFDN5 silencing (Fig. 6A). Similar alterations were found in xenograft tumors (Fig. 6B). Collectively, the findings suggest that the KDM5C-PFDN5 axis can influence the EMT activity to impact CRC progression.

## Discussion

Histone methylation is a crucial epigenetic modification involved in CRC progression (Jung et al. 2020). The histone demethylase KDM5C has been identified as playing a role in both tumor promotion and suppression depending on the specific tumor contexts and targets involved (Chang et al. 2019). In this study, the upregulation profile of KDM5C in CRC was confirmed, and its oncogenic role was attributed to increased transcriptional activity of c-Myc due to the epigenetic suppression of PFDN5.

Mutations or dysregulation of the KDM5C gene have been closely correlated with tumor growth and progression. For instance, whole-exome sequencing in human pancreatic cancers revealed truncating insertion and deletion mutations in the KDM5C gene (Wang et al. 2012). In von Hippel-Lindau-deficient clear cell renal cell carcinoma cells, the knockdown of KDM5C restored the H3K4me3 level and promoted growth of xenograft tumors in nude mice (Niu et al. 2012). The tumor promoting role of KDM5C has been observed as well. In gastric cancer, for example, KDM5C has been found to induce proliferation and invasive potential of cancer cells, partly by suppressing the tumor suppressor p53 (Xu et al. 2017). Similarly, KDM5C has been observed to accelerate growth and metastatic dissemination of lung cancer cells through the epigenetic suppression of microRNA-133a (Zhang et al. 2021). In CRC, Lin et al*.* demonstrated that KDM5C upregulation enhanced cancer cell sensitivity to chemo drugs (Lin et al. 2018). However, their subsequent research identified increased KDM5C expression in CRC cells and patients, linked to poor survival of patients. This upregulation promoted cancer cell proliferation by suppressing FBXW7 transcription and protecting the c-Jun protein from FBXW-7-mediated ubiquitination and degradation (Lin et al. 2020). These findings align with our observations that high expression of KDM5C correlates with poor survival rate and unfavorable clinical outcomes of patients. Additionally, our research revealed that the KDM5C knockdown suppressed proliferation, morbidity, and tumorigenicity of two CRC cell lines while promoting cell autophagy and apoptosis, confirming an oncogenic role of KDM5C in CRC.

Regarding the core downstream targets of KDM5C involved in the colorectal tumorigenesis, we performed bioinformatics analyses and identified PFDN5, a known binding protein negatively regulating c-Myc (Ariga 2015). An inverse correlation between KDM5C and PFDN5 was observed in CRC cells and tissues, with both KDM5C and H3K4me3 fragments enriched on the PFDN5 promoter. Not surprisingly, due to its suppressive effect on PFDN5 transcription, KDM5C increased the transcriptional activity of c-Myc. c-Myc, a well-established proto-oncogene, plays a crucial role in tumorigenic events from tumor initiation to growth and metastasis in a variety of cancers (Gan et al. 2020; Wu et al. 2020; Zhong et al. 2022). Somatic deletion of the PFDN5 gene has been observed in canine mammary cancer, particularly in solid tumors, correlating with a high Ki-67 score (tumor proliferation marker) (Hennecke et al. 2015). Yesseyeva et al*.* demonstrated that high PFDN5 mRNA expression was associated with favorable overall survival of patients with gastric cancer, which was distinct from other PFDN family members including PFDN2, PFDN3 and PFDN4 (Yesseyeva et al. 2020). However, little is known about the precise role of PFDN5 in cancer progression. Our study revealed that the suppression of proliferation, EMT. migration and invasion, and tumorigenicity of the CRC cells following KDM5C knockdown was restored upon further PFDN5 silencing. Therefore, the oncogenic function of KDM5C in CRC is, at least in part, due to the epigenetic suppression of PFDN5 and transcriptional activation of c-Myc.

Furthermore, we observed that autophagosome formation and apoptosis in CRC cells were promoted by KDM5C knockdown but suppressed by PFDN5 knockdown. Autophagy can enhance the survival of established tumor cells in the late stage. On the other hand, it can restrict tumor necrosis and the infiltration of inflammatory cells by reducing oncogene-induced senescence, suppressing the dissemination of cancer cells from the primary site (Su et al. 2015). Autophagy deficiency can lead to oxidative stress, DNA damage, and genome instability, contributing to onset and progression of cancer (Karantza-Wadsworth et al. 2007; Mathew et al. 2007), with P62 accumulation playing a major role (Mathew et al. 2009). Excessive autophagy has been linked to the induction of apoptosis, suppressing development of malignancies (Tang et al. 2021). In CRC, treatment with rapamycin, a conventional autophagy inducer, decreased cancer cell proliferation (Gulhati et al. 2009). Moreover, induction of autophagy and apoptosis has been associated with reduced lung metastasis of CRC cells (Han et al. 2019). Combining previous evidence with our findings of this paper, it can be suggested that the KDM5C/PFDN5 axis might also affect autophagy and apoptosis of cells to affect CRC progression.

## Conclusion

**Fig. 7:**
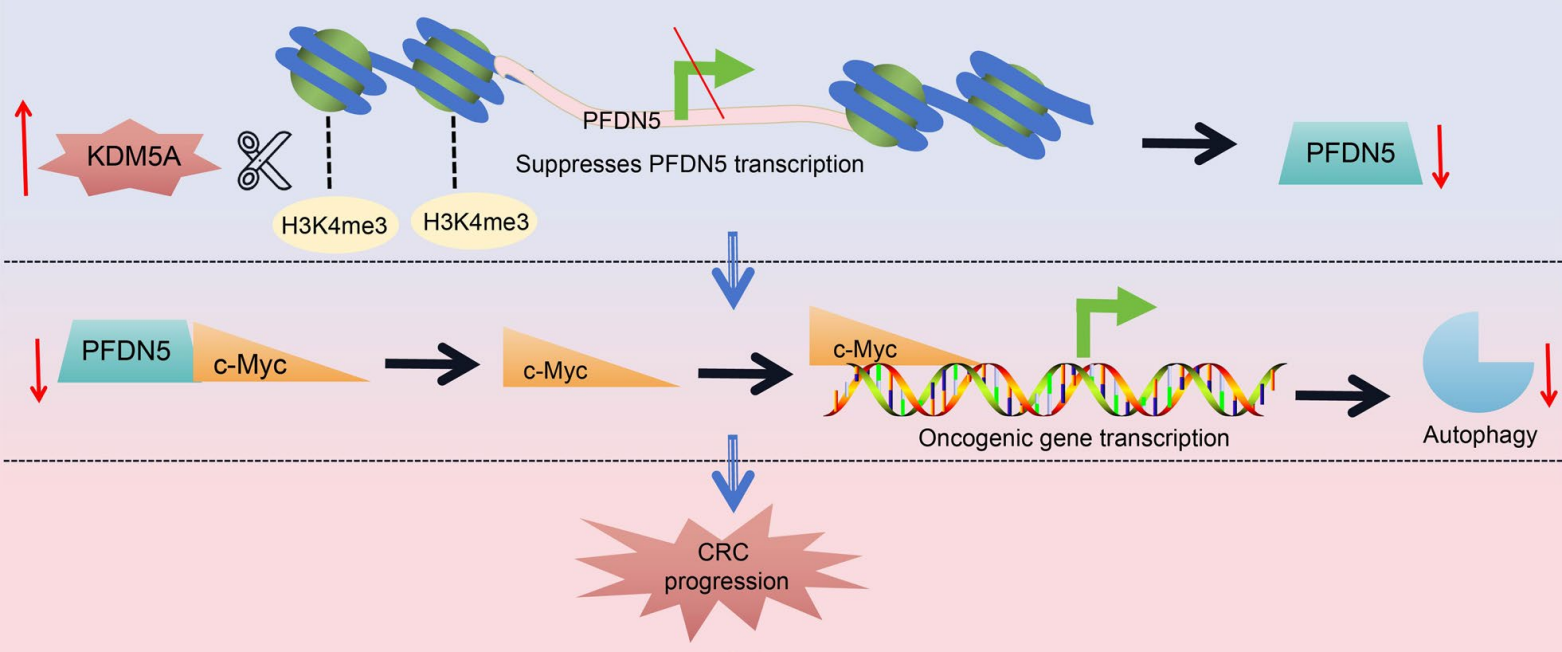
Graphical abstract. KDM5C is highly expressed in CRC. It catalyzes demethylation of H3K4me3 on the PFDN5 promoter, therefore suppressing PFDN5 transcription and leading to transcriptional activation of protooncogene c-Myc. This results in cell autophagy repression and CRC progression

In conclusion, this study compelling evidence establishing KDM5C as a tumor promoter in CRC. The major mechanism driving the oncogenic events mediated by KDM5C appears to be the epigenetic suppression of PFDN5, potentially leading to the transcriptional activation of c-Myc (Fig. 7). Functional experiments specifically exploring c-Myc were not included in the present study, given the extensive existing research on its tumorigenic role. Furthermore, our research confirmed the prognostic significance of both KDM5C and PFDN5 in CRC patients. This finding enhances the translational value of our results, suggesting that the management of CRC could benefit from strategies such as the suppression of KDM5C or the restoration of PFDN5. Development of specific pharmaceutic inhibitors of KDM5C, or gene delivery systems of PFDN5, holds promise in the treatment of CRC. Particularly, considering their roles in mediating autophagy-induced cell apoptosis, these approaches are promising options when applied alongside autophagy stimulators such as rapamycin or other mTOR inhibitors (Perez-Hernandez et al. 2019). However, the effectiveness of these approaches may vary depending on the targeted pathways and the complexity of the disease. It is essential to acknowledge that more extensive research is warranted to fully elucidate the therapeutic implications of these findings before progressing to clinical trials.

## Data Availability

Data will be made available on request.

## Abbreviations

ANOVA: Analysis of variance
ATCC: American Type Culture Collection
CCK-8: Cell counting kit-8
ChIP: Chromatin immunoprecipitation
CQ: Chloroquine
CRC: Colorectal cancer
DMEM: Dulbecco's modified Eagle's medium
FBS: Fetal bovine serum
FITC: Fluorescein isothiocyanate
GAPDH: Glyceraldehyde-3-phosphate dehydrogenase
GEPIA: Gene Expression Profiling Interactive Analysis
H3K4me3: Trimethylation of histone H3 lysine 4
HRP: Horseradish peroxidase
IgG: Immunoglobulin G
IHC: Immunohistochemistry
KDM5C: Lysine demethylase 5C
PFDN5: Prefoldin subunit 5
PI: Propidium iodide
RT-qPCR: Reverse transcription quantitative polymerase chain reaction
RFS: Recurrence-free survival
shRNA: Short hairpin RNA
WB analysis: Western blot analysis
